# Blocking primers to enhance PCR amplification of rare sequences in mixed samples – a case study on prey DNA in Antarctic krill stomachs

**DOI:** 10.1186/1742-9994-5-12

**Published:** 2008-07-20

**Authors:** Hege Vestheim, Simon N Jarman

**Affiliations:** 1 Department of Biology, University of Oslo, P.O. Box 1066, Blindern, 0316, Oslo, Norway; 2 Australian Antarctic Division, 203 Channel Highway, Kingston, TAS, 7050, Australia

## Abstract

**Background:**

Identification of DNA sequence diversity is a powerful means for assessing the species present in environmental samples. The most common molecular strategies for estimating taxonomic composition depend upon PCR with universal primers that amplify an orthologous DNA region from a range of species. The diversity of sequences within a sample that can be detected by universal primers is often compromised by high concentrations of some DNA templates. If the DNA within the sample contains a small number of sequences in relatively high concentrations, then less concentrated sequences are often not amplified because the PCR favours the dominant DNA types. This is a particular problem in molecular diet studies, where predator DNA is often present in great excess of food-derived DNA.

**Results:**

We have developed a strategy where a universal PCR simultaneously amplifies DNA from food items present in DNA purified from stomach samples, while the predator's own DNA is blocked from amplification by the addition of a modified predator-specific blocking primer. Three different types of modified primers were tested out; one annealing inhibiting primer overlapping with the 3' end of one of the universal primers, another annealing inhibiting primer also having an internal modification of five dI molecules making it a dual priming oligo, and a third elongation arrest primer located between the two universal primers. All blocking primers were modified with a C3 spacer. In artificial PCR mixtures, annealing inhibiting primers proved to be the most efficient ones and this method reduced predator amplicons to undetectable levels even when predator template was present in 1000 fold excess of the prey template. The prey template then showed strong PCR amplification where none was detectable without the addition of blocking primer. Our method was applied to identifying the winter food of one of the most abundant animals in the world, the Antarctic krill, *Euphausia superba*. Dietary item DNA was PCR amplified from a range of species in krill stomachs for which we had no prior sequence knowledge.

**Conclusion:**

We present a simple, robust and cheap method that is easily adaptable to many situations where a rare DNA template is to be PCR amplified in the presence of a higher concentration template with identical PCR primer binding sites.

## Background

*Euphausia superba *is the dominant krill species in the Antarctic and a key component of the Southern Ocean ecosystem [e.g. [1]]. The population of Antarctic krill in the Scotia Sea alone is estimated to be 208 million tonnes [2] and the krill fishery has the potential to become the world's largest fishery [3]. It has long been recognized that *Euphausia superba *feeds primarily as a herbivore during the phytoplankton-rich periods of spring and summer, at least during daytime [e.g. [4], although heterotrophic food items are also known to be consumed [5]]. However, there is uncertainty about how adult Antarctic krill survive the winter. A variety of strategies have been proposed since krill do not seem to build up large fat reserves. These strategies include shrinkage [e.g. [6,7]], lowered metabolic rates [8,9], switching to omnivory [10] or feeding on ice associated biota [e.g. [11]].

Krill diet is difficult to fully characterise because they feed on a very wide taxonomic range of prey items. The diversity of prey consumed by krill means that many dietary analysis methods will produce biased results because of the difference in detectability of different prey items. As a result of this, a number of methods for analysing krill diet have been tried.

DNA-based methods are perhaps the most promising ones and are now established as powerful tools for studying food chains [12]. Their advantage lies in enabling identification of prey when the remains are degraded or lack hard parts. As species-specific DNA sequences are moderately easy to identify, these methods are also generally better at making species-level identifications than other biomarker methods such as stable isotopes, signature lipids and antigen detection [13]. However, the predominance of one DNA template within a single sample can bias or restrict molecular analysis [14,15]. This is often the case in diet studies since prey DNA in stomach- and faecal samples tend to be far more degraded than predator DNA. The extent of this overabundance of amplifiable predator DNA increases with the size of the target fragment [16,17].

PCR (polymerase chain reaction)-based methods using universal primers will not be sensitive enough to detect low-abundance sequences since rare prey templates have a tendency for being missed in the early stages of the PCR. The universal primers which are conserved among the target prey species usually also amplify DNA from the predator by necessity, and hence prey-DNA is masked by predator-DNA [18,19]. Several approaches have therefore been applied in order to avoid only detecting the predator-DNA.

**Figure 1:**
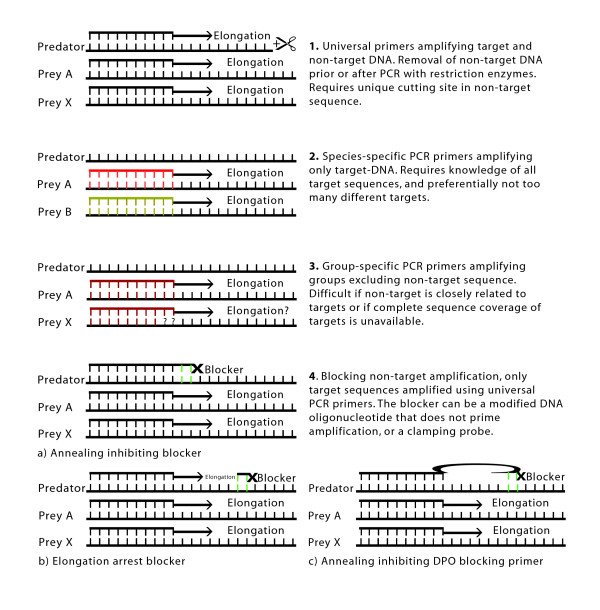
**Screening methods**. Schematic illustration of different ways to screen away a dominating DNA template in a PCR mixture. 1. Universal primers amplifying target and non-target DNA and removal of non-target DNA prior or after PCR with restriction enzymes. 2. Species-specific PCR primers amplifying only target-DNA. 3. Group-specific PCR primers amplifying groups excluding non-target sequence. 4. Blocking non-target amplification, only target sequences amplified using universal PCR primers a) annealing inhibiting blocking primer, b) elongation arrest blocking primer c) annealing inhibiting DPO blocking primer.

One of these methods is to remove predator DNA after PCR amplification by restriction enzymes [e.g. [20,21]; Figure 1]. But this method requires a unique cutting site in the predator sequence, which is often difficult to find. Furthermore, it will not work if the PCR has failed to amplify prey DNA because the predator DNA has dominated the PCR totally. A method (the SuPER-method) to cut only target DNA in a mix prior to PCR has been applied by Green and Minz [15] investigating microbial diversity, but this method still requires several extra handling steps and has apparently not achieved wide acceptance.

Another common method is to design species specific primers or probes for the detection of prey species of interest [e.g. [22-24]; Figure 1]. This approach is however not suitable if the potential range of prey species is large (given the need to design perhaps hundreds of probes) or unknown (making it impossible to design prey specific probes *a priori*). This is clearly the case with krill diet. It is also possible to design group specific primers targeting broader groups of potential prey, but not the predator [e.g. [25]]. This approach has previously been applied to studying the diatom component of Antarctic krill diet [26]. However, developing group-specific primers is problematic if they are designed for groups where complete sequence coverage is not available. In such situations the group specific primers might screen away not only predator DNA but also DNA from prey species of interest.

One way to avoid only detecting predator DNA, and at the same time ensuring that no potential prey is screened away, is to use universal PCR primers accompanied by a method that specifically blocks predator DNA from amplification.

"PCR-clamping" where peptide nucleic acids (PNAs), locked nucleic acids (LNAs), or morpholinos are used to suppress PCR amplification of wild-type or dominant sequences is one such method [see [27] for a recent review]. PNA/DNA duplexes for example have a higher melting temperature compared with DNA/DNA duplexes [28] and do not prime DNA polymerisation [29]. Hence, a sequence-specific PNA probe overlapping one of the PCR-primer attachment sites within the target sequence of interest will not work as a primer. This method has been widely applied to minimize amplification of the dominant sequence in the fields of clinical chemistry [e.g. [30]], environmental microbiology [e.g. [31]], parasitology [32], and to detect genetically modified organism content in food [33], but not to our knowledge in diet studies. However, synthesis time for clamping probes is several weeks and these probes are also quite expensive.

A simpler method compared to PCR-clamping is to use a predator specific blocking primer (i.e. a DNA oligo that binds to predator DNA by preference but is modified so that it does not prime amplification) (Figure 1). This primer can compete with universal primers in a mix and block predator DNA amplification. Blocking primers have also been demonstrated to be useful in clinical research [e.g. [34]] and environmental microbiology studies, reducing amplification of a bacterial sequence that otherwise would have dominated the clone library [35]. In this study we test the application of blocking primers in diet studies and apply the method to assessing the diet of Antarctic krill caught during the austral autumn and winter.

## Methods

### Primer design

**Figure 2:**
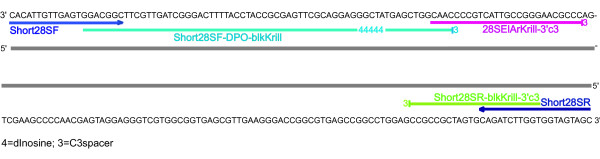
**Primers**. A 203 bp region of *Euphausia superba *28S sequence showing the location of the different primers applied in this study.

Primers applied in analysing of gut content of predators should ideally target short sequences of multiple-copy DNA because of the degraded nature of the prey derived sequences [13,16,17,36]. The ribosomal DNA is therefore often used as a target for PCR amplification in diet studies because ribosomal DNA (rDNA) genes are repeated tandemly in high copy numbers and are highly conserved within species [37]. We designed 'universal' PCR primers that amplify a short, but fairly variable region of the 28S rDNA from all eukaryotes tested. We also designed three blocking primers intended to bind to the *Euphausia superba *sequence amplified by the universal primers. The primers used in this study are given in Table 1 and shown aligned with the krill sequence in Figure 2.

**Table 1:** 28S PCR primers used in this study.

Primer name	Sequence (5' → 3')
Short28SF	GTGTAACAACTCACCTGCCG
Short28SR	GCTACTACCACCAAGATCTG
Short28SseqF	AGCAGGACGGTGGYCATGGAAGTCG
Short28SseqR	GCACTGGGCAGAAATCACATTGCG
28S-ElArKrill-3'c3	GTTGGGGCAGTAACGGCCCTTGCGGG3
Short28SR-blkKrill-3'c3	CCACCAAGATCTGCACTAGCGGCGG3
Short28SF-DPO-blkKrill	CCTGCCGAAGCAACTAGCCCTGAAAATGGATG GCGCTCAAGCGTCCTC44444ACTCGACCGTTG3

	4 = dInosine; 3 = C3spacer

The blocking primer, 'Short28SR-blkKrill3'c3' overlapped with the 3' end of the reverse universal primer, but extended into krill-specific sequence and was modified with a C3 spacer at the 3'-end (Figure 2). We needed a modification which was 100% synthesized (i.e. no oligos missing it) and which was stable (i.e. no degradation or enzymatic removing of the modification after synthesis). Even if just a small percentage of the blocking primers were to prime amplification of predator DNA as a result of not having the 3' modification, this would render the procedure unworkable. C3 spacer (3 hydrocarbons) CPG is a standard primer modification available from most suppliers of custom oligonucleotides. Adding this modification to the 3'-end of an oligonucleotide prevents elongation during a PCR without noticeably influencing its annealing properties. Because oligos are synthesized from a 3' to 5' direction, all molecules will be modified with the 3' modification. Modifying the 3'- end with a phosphate group (as chosen by Liles et al. [35]), a phosphate ester, or using an inverted 3'-3' linkage would also prevent elongation. However, side reactions during deprotection of the oligonucleotide or enzymatic impurities may free the 3'-hydroxyl group to a small extent, and these methods are not so effective in blocking as C3 spacers CPG [38,39].

Because finding an appropriate binding site for a species specific primer next to a binding site of a universal primer is often difficult, a krill specific blocking primer, Short28SF-DPO-blkKrill overlapping with the 3'end of the forward universal primer and having an internal modification of five deoxyinosine (dI) molecules in addition to the C3 spacer modification was also designed (Figure 2). Very long conventional oligonucleotides often do not work. In general, primers longer than 25 bases are rarely used since their Tms can be over 70°C, which is too high for effective PCR cycling [40]. Long primers also often generate many non-specific bands resulting from non-specific annealing. A dual priming oligonucleotide (DPO) [41] contains two separate priming regions joined by a polydeoxyinosine linker. DPOs does not suffer from the limitations of a high Tm since the linker assumes a bubble-like structure resulting in two primer segments with distinct annealing properties. Furthermore, the bubble-like structure of linker efficiently prevents primer-dimer and hairpin structure formation [41].

The Short28SR-blkKrill3'c3 and the Short28SF-DPO-blkKrill blocking primers were both designed to prevent annealing of the unmodified version of the universal primer on krill sequences. Further a third krill specific blocking primer situated between the two universal primers was tested (Figure 2). This was an "elongation arrest" primer ([42]; Figure 1) and also had a C3 spacer at its 3' end.

### Sampling and DNA extraction

Total genomic DNA was extracted from ethanol-preserved specimens of Antarctic pelagic invertebrates or cultured algal species (Table 2).

**Table 2:** List of species used for DNA analysis in this study with sampling site and GenBank accession numbers.

Species	Location/ Cruise	Position	Date	Time start (and stop) (UTC)		GenBank accession number
Algae						
*Pyramimonas *sp.	Ace Lake, Vestfold Hills	68° 28' 18.5	S 78° 11' 16.1" E			EU375499
Chaetognatha						
*Sagitta gazellae*	SIPEX	65 03.81 S, 119 41.91 E	29/09/2007	11:10		EU375500
Copepoda						
*Calanus* *propinquus*	SIPEX	64 13.94 S 116 33.86 E	08/10/2007	6:46		EU375501
*Euchirella* *rostromagna*	SIPEX	65 03.81 S, 119 41.91 E	29/09/2007			EU375502
*Metridia* *gerlachei*	SIPEX	64 54.721 S 177 1376 E	05/10/2007	13:14		EU375503
*Pareuchaeta* *pseudotonsa*	SIPEX	65 03.81 S, 119 41.91 E	29/09/2007			EU375504
Amphipoda						
*Primno* *macropa*	SIPEX	65 03.81 S, 119 41.91 E	29/09/2007	11:10		EU375505
*Themisto* *gaudichaudi*	SIPEX	65 03.81 S, 119 41.91 E	29/09/2007	11:10		EU375506
Krill stomachs						
	V4 06/07	66 04 27 S, 109 58 95 E	24/03/2007	08:51–08:56	0–100 m	EU378965-EU379000
	SIPEX	65 18 622 S, 125 37 353 E	17/09/2007	12:00	16–60 m	
	SIPEX	65 28 04 6 S, 120 39 92 6 E	20/09/2007	12:37–12:57	0–200 m	

Krill were collected during two cruises in eastern Antarctic waters with RSV *Aurora Australis*. The first cruise took place during the austral autumn and the second in late austral winter (Table 2). Krill were collected using Rectangular Midwater Trawl-8 net by standard double oblique tows from the surface down to 200 m or by target towing at aimed depths of 100 m or 16–60 m (Table 2). The vessel speed during net towing was maintained at 2 knots. Immediately after capture, krill were put into jars containing 80% ethanol as recommended by Passmore et al. [26].

After 1–6 months storage krill stomachs (foreguts) of ethanol rinsed krill were dissected under a dissecting microscope in sterile Petri dishes using flame sterilized forceps. Care was taken so the stomach was not in contact with any outer parts of the krill's exoskeleton. The stomach was then briefly rinsed in fresh ethanol and put into an autoclaved DNA free Eppendorf tube filled with ATL buffer (Qiagen). The sample was homogenized using a sterile plastic pestle prior to DNA extraction.

All DNA was extracted with a DNeasy Blood & Tissue kit (Qiagen) following the manufacturer's instruction for animal tissue and total DNA yield and quality of final extractions (100 μL) was determined using Picofluor 8000-004 (Turner Designs) and samples stained with Quant-iT PicoGreen ds DNA reagent (Molecular Probes).

### Testing of blocking primers

To evaluate the efficiency of the different types and different amounts of blocking primers, artificial rDNA mixtures were created. First PCR amplification were performed on krill and *Pyramimonas *DNA with the universal 28S rDNA primers Short28SF and Short28SR (Table 1) as 25 μL reactions with 0.25 μL of each oligo (10 μM), 0.25 μL dNTP, 2.5 μL 10×, 0.1 μL Platinum *Taq *DNA Polymerase High Fidelity (Invitrogen) 5 U^.^ μL^-1^, 1 μL Mg^2+ ^and 5 μL DNA (~10 ng^.^ μL^-1^).

The PCR products were then cloned using the TOPO TA Cloning system competent cells (Invitrogen). Transformants were screened using blue/white selection on LB-agar containing X-Gal and 10 mg^.^mL^-1 ^ampicillin or kanamycin. White or light blue colonies were picked for plasmid isolation, reincubated overnight in LB medium containing antibiotics and plasmids were then extracted using the Ultraclean miniplasmid extraction kit (Mo Bio, Carlsbad, CA, USA). Plasmids were linearized using *Hind*III restriction enzyme and the yield quantified using Picofluor 8000-004 (Turner Designs) and samples stained with Quant-iT PicoGreen ds DNA reagent (Molecular Probes).

Samples were thus mixed to contain a 100-fold and a 1000-fold excess of target rDNAs (krill 28SrDNA) compared with nontarget rDNAs (*Pyramimonas *sp.). A sample containing only krill DNA was used as a control.

Amplification with blocking primers were performed as 25 μL reactions with 0.25 μL of each of the universal 28S rDNA primers Short28SF(10 μM) and Short28SR(10 μM), 0.25 μL dNTP, 2.5 μL 10×, 0.1 μL Platinum *Taq *DNA Polymerase High Fidelity (Invitrogen) 5 U^.^ μL^-1^, 1 μL Mg^2+ ^and a variable amount of blocking primer and rDNA. PCR thermal cycling conditions were: 2 min at 94°C; 40 cycles of 10 s at 94°C, 30 s at 59°C, 30 s at 68°C; and finally 5 min at 72°C.

Blocking efficiency was assessed by fragment analysis of the fluorescently labelled PCR products. Fragment analysis separates a mixture of DNA fragments according to their sizes and is much more sensitive than standard gel electrophoresis. The analysis was performed on an Applied Biosystems 3130 xl Capillary Electrophoresis (CE) Genetic Analyser and results were analyzed with Peak Scanner Software 1.0 (Applied Biosystems).

### PCR amplification of prey DNA from krill stomachs

After determining the most efficient blocking primer mixture, PCR was performed on krill stomach isolates. PCRs were prepared using UV sterilized equipment and consumables and negative (no-template) controls were always run alongside the samples. The PCR reactions were carried out on a MJ Research DNA engine Gradient Cycler (eq. Chromo 4) and sequencing was performed on a 3730 xl DNA analyzer (Applied Biosystems). PCR products were checked by electrophoresis on a 1.3% agarose gel stained with SYBR^®^Safe DNA gel stain (Invitrogen). Visible bands were cut out and purified with Bio-Rad Quantum Prep Freeze'N Squeeze DNA Gel Extraction Spin Columns.

The PCR products were thereafter reincubated for 10 min at 72°C with 20 μL reaction volume containing 2 μL 10×, 1 μL Mg^2+^, 0.2 μL Biotaq TM DNA Polymerase (Bioline) and 0.2 μL dNTP to add 3' adenines and cloned using the TOPO TA Cloning system competent cells (Invitrogen). Transformants were picked for PCR and amplification was performed with the TOPO_F and TOPO_R primers as 25 μL reactions with 0.25 μL of each oligo (10 μM), 0.25 μL dNTP, 2.5 μL 10×, 0.1 μL Platinum *Taq *DNA Polymerase High Fidelity (Invitrogen) 5 U^.^ μL^-1 ^and 1 μL Mg^2+^. PCR thermal cycling conditions were: 2 min at 94°C; 40 cycles of 10 s at 94°C, 30 s at 65°C, 30 s at 68°C; and finally 5 min at 72°C.

Sequences were generated from these PCR products with the M13 forward primer and BigDye Terminator v3.1 sequencing reactions (ABI).

Amplification for sequencing of species not yet available in GenBank were performed as 25 μL reactions with 0.25 μL of each of the universal 28S rDNA primers Short28SseqF and Short28SseqR (10 μM) (Table 1) designed to cover a larger area than the Short28SF/Short28SR primers, including their primer binding sites, 0.25 μL dNTP, 2.5 μL 10×, 0.1 μL Platinum *Taq *DNA Polymerase High Fidelity (Invitrogen) 5 U^.^ μL^-1 ^and 1 μL Mg^2+^. PCR thermal cycling conditions were unmodified except for increasing the elongation step to 60 s.

### Clone identification

Clones were tentatively identified by finding their closest match in the GenBank database using the BLASTN algorithm [43]. All the sequences (clones and closest matches) were then aligned in MEGA 4 [44] and a similarity tree was created using Tamura-Nei distances [45] and the minimum evolution algorithm with gap handling by pairwise deletion.

## Results

### Blocking primer performance

Adding a blocking primer to the PCR mixture clearly decreased the number of predator fragments amplified and enriched the number of rarer prey fragments. In rDNA mixtures containing 1000 times as many "predator" (krill) rDNA fragments compared to "prey" (algae) rDNA fragments, only peaks corresponding to krill rDNA could be detected by fragment analysis when no blocking primer was added (Table 3). Adding an annealing inhibiting blocking primer in a ratio 4:1 compared to the corresponding universal primer (1.0 μL blocking primer and 0.25 μL universal primer, both 10 μM) led to reduced amplification of krill rDNAs but not to complete amplification arrest (Table 3). And by adding 10 times as much blocking primer as universal primers (2.5 μL blocking primer and 0.25 μL each universal primer), algal rDNA was almost exclusively amplified (Table 3). Adding 20 times as much blocking primer (5.0 μL blocking primer and 0.25 μL each universal primer) decreased the krill peak even further (Table 3).

**Table 3:** Results from fragment analysis showing the algae and krill peak height generated from PCR mixtures with different amounts of the different blocking primers added and different initial concentration of algal 28S rDNA fragments.

Initial concentration of *Pyramimonas *fragments to *Euphausia *fragments	Blocking primer	Amount of 10 μM blocking primer added	Peak height	
			
			Fragment length 180 (*Pyramimonas *sp.)	Fragment length 199 (*Euphausia superba*)
1:100	No blocking	0 μL	311	8107
				
	Short28SR-	1.0 μL	8454	3030
		2.5 μL	8516	186
	blkKrill-3'c3	5.0 μL	8243	0
				
	Short28SF-	1.0 μL	8374	7538
		2.5 μL	8524	190
	DPO-blkKrill	5.0 μL	8498	66
				
1:1000	No blocking	0 μL	0	8301
				
	Short28SR-	1.0 μL	8480	6025
		2.5 μL	8470	146
	blkKrill-3'c3	5.0 μL	8617	0
				
	Short28SF-	1.0 μL	7740	8309
		2.5 μL	8427	318
	DPO-blkKrill	5.0 μL	8427	140
				
Krill only	No blocking	0 μL	0	8324

The "normal" primer Short28SR-blkKrill3'c3 seemed to be somewhat more efficient in blocking predator DNA compared to the DPO primer Short28SF-DPO-blkKrill. Peaks corresponding to krill rDNA were lower in the 4:1 blocking primer: universal primer mixtures for both the 1:100 and the 1:1000 prey: predator rDNA samples with the normal primer compared to the mixtures with the DPO primer (Table 3). However, the DPO primer largely blocked krill DNA amplification when added in a ratio of 10:1 to the unmodified primer (Table 3).

The elongation arrest primer 28S-ElArKrill-3'c3 did not work. No PCR product at all was generated when it was added to the PCR mixture (data not shown).

### Krill stomach analysis

**Figure 3:**
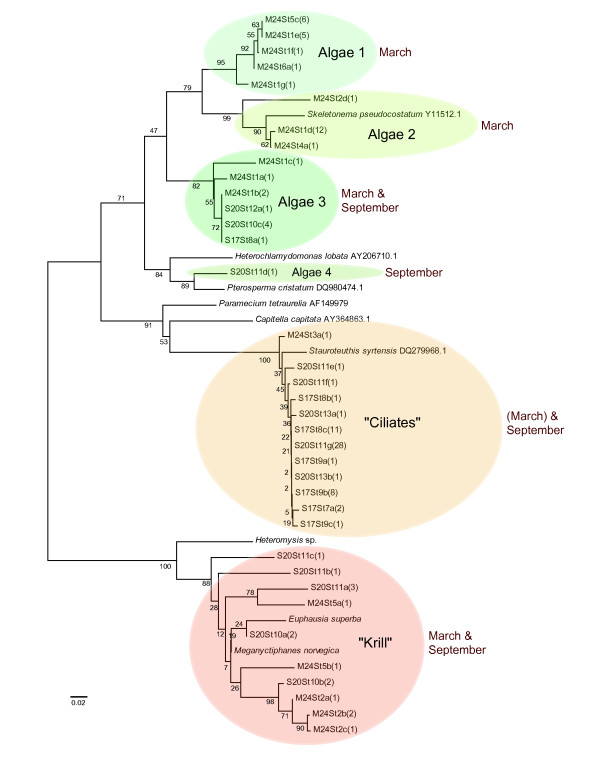
**Sequence similarity tree**. Sequence similarity tree of sequences amplified from krill stomachs and related sequences. The krill stomach sequences are named so as the first three letters means sampling date, i.e. M24 = March 24. 2007, S17 and S20 = September 17. and September 20. 2007, St. means Stomach number and the number in parenthesis equals the number of identical sequences of each different sequence found in the different stomachs. The tree is unrooted.

In total 111 clones from 13 different krill stomachs were sequenced revealing 36 different sequences falling into 6 major groups. Among them there were no sequences corresponding to the wild type of krill 28s rDNA. 10 different (15 in total) sequences were however clearly of krill origin (Figure 3) and probably pseudogenes or low copy versions normally not detected by direct sequencing.

Four different groups of algal sequences were found. Two of these groups, only detected in the March samples, had their closest match to species belonging to Bacillariophyta, respectively the genus *Phaeodactylum *and the genus *Skeletonema *(Figure 3). A third group, found in stomachs from all three sampling dates seemed to be somewhat similar to the Bacillariophyta, but did not match anything in GenBank (Figure 3). The 200 bp area sequenced was too uninformative to reveal where the group belonged by phylogentic analysis, besides that the group most likely was of algal origin. The last algal group was one sequence from September belonging to Chlorophyta (Figure 3).

One large group of sequences, which was prevalent in the September sample, could not be unequivocally identified by reference to current sequences in GenBank. The closest matches was a 96% match to *Stauroteuthis *(Mollusca). It is possible that this may represent mollusc food items such as larval squid. However, it is also possible that the *Stauroteuthis *sequence has been misreported and is actually derived from a contaminant of the original *Stauroteuthis *sample.

The sequences have been deposited to GenBank under the accession numbers EU378965 – EU379000.

## Discussion

This study shows that complete removal of predator sequences could be achieved by adding a predator specific blocking primer to a PCR mixture. Even though more sophisticated sequencing techniques such as pyrosequencing [46] and polony sequencing [47] now open new possibilities in assessing sequence diversity of mixed templates [48], applying a method that screens away templates assumed to occur in 1000-fold excess compared to what you are looking for will certainly be beneficial. Adding a predator specific blocking primer is also a simple method that allows for using a single PCR to simultaneously amplify DNA from all prey items present in each stomach sample. Furthermore, it does not depend on tests being developed *a priori *for potential prey, the only sequence that is needed to be known, is the sequence of the predator itself.

In this study we also show that a DPO primer [41] having the 3' end blocked also could be used as a blocking primer. This extends the region to look for species-specific blocking sites and is very useful especially since initial trials with a krill specific 'elongation arrest' primer located between the forward and reverse universal primer did not work.

It is not known why the elongation arrest primer was unsuccessful. Von Wintzingerode et al. [31] and also Peano et al. [33] using PCR clamping and PNA probes found that elongation arrest clamping worked, but that competitive clamping (annealing inhibition) was more efficient, and the former hypothesised that this was due to interaction between the *Taq *polymerase and PNA and DNA. However, since elongation arrest primers allows the unmodified version of the universal primer to bind to the dominating sequence, and just inhibit the generating of its amplicons, perhaps the explanation of no PCR product is that the universal primers never found the rarer sequences. It is also a possibility that the 'blocking primer' of Lewis et al. [42] is simply producing a more efficient PCR reaction with a shorter product, rather than truly 'blocking' the amplification of the longer product.

Blocking primer performance was sensitive to the amount of blocking primer added. We found that a 10 fold molar excess was sufficient for complete blocking to the detection limit of our assay, when the dominant template occurred in 1000 fold excess of the minor template. It is difficult to compare primer concentrations between blocking primer studies since concentration of the unmodified primers and DNA templates also tends to vary (like e.g. vs. reference [34]). The blocking primer in our study was designed to be species specific. However, it could block other species as well, something that cannot be excluded without extensive empirical testing. Keeping blocking primer concentration at the minimum required amount is probably a good rule of thumb.

We only identified algal sequences in the krill stomachs besides krill pseudogenes/low copy genes and an anonymous phylotype. Complete sequence coverage of all the potential krill prey was not available and it was naturally not possible to test experimentally if the blocking primers designed to be krill specific also blocked any other organisms beside krill. However, since krill obviously could and did feed on algae at both occasions, the DNA types detected were probably representative snapshots of krill diet at the three different sampling times.

Krill caught in September differed from krill caught in March in that sequences belonging to Bacillariophyta (diatoms) only were detected in the March samples. Further, a sequence belonging to Chlorophyta was only found in krill caught in September. The unidentified algal group, found in stomachs from all three sampling dates seemed to be related to diatoms, even though it is difficult to determine based on a 200 bp fragment. Many known Antarctic algal groups, including the frequently dominant group Parmales that is morphologically similar to diatoms [49], are not yet represented in sequence databases because they are so difficult to culture. Our unidentified algal group could belong to Parmales, or be something else. We also found another unknown phylotype. Finding rDNA that does not match any known sequences is common in molecular studies of eukaryotic diversity [e.g. [19,50]]. But as barcoding initiatives results in more and more available sequence data, especially if they could include also other DNA regions beside mtCOI, this will be less of a problem in the future.

The identification of several krill pseudogenes or low copy genes is interesting. Different strategies are being developed in analyzing functional RNA genes [51]. Indicators of pseudo-rDNA genes might for example be elevated levels of uncompensated base changes within stem regions (see e.g. [52]). We did not perform secondary structure predictions for the 'krill-like' sequences, and cannot tell whether they are pseudogenes or expressed and functional low-copy versions. 28S rDNA is expected to be very homogenized by molecular drive [53], but there are also reports of distinct rDNA classes within species [52,54]. Whatever is the case with the sequences detected here, the results demonstrate that by blocking dominant sequences from amplification, interesting variation is revealed that might remain undetected by conventional PCR-cloning assays.

## Conclusion

DNA based techniques are valuable in tracking predator-prey interactions because they do not rely on the presence of visually diagnostic remains. In this study we have demonstrated how blocking primers can be used to filter out the dominating DNA from the predator itself, allowing detection of food derived DNA fragments by the use of universal PCR primers.

## Competing interests

The authors declare that they have no competing interests.

## Authors' contributions

HV carried out most of the laboratory work and data analysis and drafted the manuscript. SNJ designed the study and did some of the preliminary laboratory work. Both authors participated in the development of concepts presented in the paper and contributed significantly to the writing of the final version of the manuscript. Both authors read and approved the final manuscript.

## References

[B1] Loeb V, Siegel V, HolmHansen O, Hewitt R, Fraser W, Trivelpiece W, Trivelpiece S (1997). Effects of sea-ice extent and krill or salp dominance on the Antarctic food web. Nature.

[B2] Heywood BG, Brierley AS, Gull SF (2006). A quantified Bayesian maximum entropy estimate of Antarctic krill abundance across the Scotia Sea and in small-scale management units from the CCAMLR-2000 survey. CCAMLR Science.

[B3] Croxall JP, Nicol S (2004). Management of Southern Ocean fisheries: global forces and future sustainability. Antarctic Science.

[B4] Marr JSW (1962). Discovery report.

[B5] Schmidt K, Atkinson A, Petzke KJ, Voss M, Pond DW (2006). Protozoans as a food source for Antarctic krill,* Euphausia superba*: Complementary insights from stomach content, fatty acids, and stable isotopes. Limnology and Oceanography.

[B6] Ikeda T, Dixon P (1982). Body shrinkage as a possible over-wintering mechanism of the Antarctic krill, *Euphausia superba *Dana. Journal of Experimental Marine Biology and Ecology.

[B7] Nicol S, Stolp M, Cochran T, Geijsel P, Marshall J (1992). Growth and shrinkage of Antarctic krill *Euphausia superba* from the Indian Ocean sector of the Southern Ocean during summer. Marine Ecology-Progress Series.

[B8] Kawaguchi K, Matsuda O, Ishikawa S, Naito Y (1986). A light trap to collect krill and other micronektonic and planktonic animals under the Antarctic coastal fast ice. Polar Biology.

[B9] Quetin LB, Ross RM (1991). Behavioral and physiological characteristics of the Antarctic krill, *Euphausia superba*. American Zoologist.

[B10] Lancraft TM, Hopkins TL, Torres JJ, Donnelly J (1991). Oceanic micronektonic macrozooplanktonic community structure and feeding in ice covered Antarctic waters during the winter (ameriez 1988). Polar Biology.

[B11] Stretch JJ, Hamner PP, Hamner WM, Michel WC, Cook J, Sullivan CW (1988). Foraging behavior of Antarctic krill *Euphausia superba* on sea ice microalgae. Marine Ecology-Progress Series.

[B12] King RA, Read DS, Traugott M, Symondson WOC (2008). Molecular analysis of predation: a review of best practice for DNA-based approaches. Molecular Ecology.

[B13] Symondson WOC (2002). Molecular identification of prey in predator diets. Molecular Ecology.

[B14] Polz MF, Cavanaugh CM (1998). Bias in template-to-product ratios in multitemplate PCR. Appl Environ Microbiol.

[B15] Green SJ, Minz D (2005). Suicide polymerase endonuclease restriction, a novel technique for enhancing PCR amplification of minor DNA templates. Applied and Environmental Microbiology.

[B16] Nejstgaard JC, Frischer ME, Simonelli P, Troedsson C, Brakel M, Adiyaman F, Sazhin AF, Artigas LF (2008). Quantitative PCR to estimate copepod feeding. Marine Biology.

[B17] Deagle BE, Eveson JP, Jarman SN (2006). Quantification of damage in DNA recovered from highly degraded samples – a case study on DNA in faeces. Front Zool.

[B18] Deagle BE, Tollit DJ, Jarman SN, Hindell MA, Trites AW, Gales NJ (2005). Molecular scatology as a tool to study diet: analysis of prey DNA in scats from captive Steller sea lions. Molecular Ecology.

[B19] Jarman SN, Deagle BE, Gales NJ (2004). Group-specific polymerase chain reaction for DNA-based analysis of species diversity and identity in dietary samples. Molecular Ecology.

[B20] Blankenship LE, Levin LA (2007). Extreme food webs: Foraging strategies and diets of scavenging amphipods from the ocean's deepest 5 kilometers. Limnology and Oceanography.

[B21] Blankenship LE, Yayanos AA (2005). Universal primers and PCR of gut contents to study marine invertebrate diets. Molecular Ecology.

[B22] Agusti N, Shayler SP, Harwood JD, Vaughan IP, Sunderland KD, Symondson WOC (2003). Collembola as alternative prey sustaining spiders in arable ecosystems: prey detection within predators using molecular markers. Molecular Ecology.

[B23] Nejstgaard JC, Frischer ME, Raule CL, Gruebel R, Kohlberg KE, Verity PG (2003). Molecular detection of algal prey in copepod guts and fecal pellets. Limnology and Oceanography: Methods.

[B24] Vestheim H, Edvardsen B, Kaartvedt S (2005). Assessing feeding of a carnivorous copepod using species-specific PCR. Marine Biology.

[B25] Jarman SN, Redd KS, Gales NJ (2006). Group-specific primers for amplifying DNA sequences that identify Amphipoda, Cephalopoda, Echinodermata, Gastropoda, Isopoda, Ostracoda and Thoracica. Molecular Ecology Notes.

[B26] Passmore AJ, Jarman SN, Swadling KM, Kawaguchi S, McMinn A, Nicol S (2006). DNA as a dietary biomarker in Antarctic krill, *Euphausia superba*. Marine Biotechnology.

[B27] Karkare S, Bhatnagar D (2006). Promising nucleic acid analogs and mimics: characteristic features and applications of PNA, LNA, and morpholino. Applied Microbiology and Biotechnology.

[B28] Egholm M, Buchardt O, Christensen L, Behrens C, Freier SM, Driver DA, Berg RH, Kim SK, Norden B, Nielsen PE (1993). PNA hybridizes to complementary oligonucleotides obeying the Watson-Crick hydrogen-bonding rules. Nature.

[B29] Ørum H, Nielsen PE, Egholm M, Berg RH, Buchardt O, Stanley C (1993). Single-base pair mutation analysis by PNA directed PCR clamping. Nucleic Acids Research.

[B30] Hancock DK, Schwarz FP, Song FH, Wong LJC, Levin BC (2002). Design and use of a peptide nucleic acid for detection of the heteroplasmic low-frequency mitochondrial encephalomyopathy, lactic acidosis, and stroke-like episodes (MELAS) mutation in human mitochondrial DNA. Clinical Chemistry.

[B31] von Wintzingerode F, Gobel UB, Stackebrandt E (1997). Determination of microbial diversity in environmental samples: pitfalls of PCR-based rRNA analysis. Fems Microbiology Reviews.

[B32] Troedsson C, Lee RF, Walters T, Stokes V, Brinkley K, Naegele V, Frischer ME (2008). Detection and discovery of crustacean parasites in blue crabs (Callinectes sapidus) by 18S rDNA targeted denaturing high-performance liquid chromatography (DHPLC). Appl Environ Microbiol.

[B33] Peano C, Lesignoli F, Gulli M, Corradini R, Samson MC, Marchelli R, Marmiroli N (2005). Development of a peptide nucleic acid polymerase chain reaction clamping assay for semiquantitative evaluation of genetically modified organism content in food. Analytical Biochemistry.

[B34] Khanna M, Park P, Zirvi M, Cao WG, Picon A, Day J, Paty P, Barany F (1999). Multiplex PCR/LDR for detection of K-ras mutations in primary colon tumors. Oncogene.

[B35] Liles MR, Manske BF, Bintrim SB, Handelsman J, Goodman RM (2003). A census of rRNA genes and linked genomic sequences within a soil metagenomic library. Applied and Environmental Microbiology.

[B36] Zaidi RH, Jaal Z, Hawkes NJ, Hemingway J, Symondson WOC (1999). Can multiple-copy sequences of prey DNA be detected amongst the gut contents of invertebrate predators?. Molecular Ecology.

[B37] Woese CR (1987). Bacterial evolution. Microbiological Reviews.

[B38] Dames S, Margraf RL, Pattison DC, Wittwer CT, Voelkerding KV (2007). Characterization of aberrant melting peaks in unlabeled probe assays. Journal of Molecular Diagnostics.

[B39] Cradic KW, Wells JE, Allen L, Kruekeberg KE, Singh RJ, Grebe SKG (2004). Substitution of 3'-phosphate cap with a carbon-based blocker reduces the possibility of fluorescence resonance energy transfer probe failure in real-time PCR assays. Clinical Chemistry.

[B40] Wu DY, Ugozzoli L, Pal BK, Qian J, Wallace RB (1991). The effect of temperature and oligonucleotide primer length on the specificity and efficiency of amplification by the polymerase chain-reaction. DNA Cell Biol.

[B41] Chun JY, Kim KJ, Hwang IT, Kim YJ, Lee DH, Lee IK, Kim JK (2007). Dual priming oligonucleotide system for the multiplex detection of respiratory viruses and SNP genotyping of CYP2C19 gene. Nucleic Acids Research.

[B42] Lewis AP, Sims MJ, Gewert DR, Peakman TC, Spence H, Crowe JS (1994). Taq DNA-polymerase extension of internal primers blocks polymerase chain-reactions allowing differential amplification of molecules with identical 5'-end and 3'-end. Nucleic Acids Research.

[B43] Altschul SF, Madden TL, Schaffer AA, Zhang JH, Zhang Z, Miller W, Lipman DJ (1997). Gapped BLAST and PSI-BLAST: a new generation of protein database search programs. Nucleic Acids Research.

[B44] Tamura K, Dudley J, Nei M, Kumar S (2007). MEGA4: Molecular evolutionary genetics analysis (MEGA) software version 4.0. Molecular Biology and Evolution.

[B45] Tamura K, Nei M (1993). Estimation of the number of nucleotide substitutions in the control region of mitochondrial DNA in humans and chimpanzees. Mol Biol Evol.

[B46] Margulies M, Egholm M, Altman WE, Attiya S, Bader JS, Bemben LA, Berka J, Braverman MS, Chen YJ, Chen ZT, Dewell SB, Du L, Fierro JM, Gomes XV, Godwin BC, He W, Helgesen S, Ho CH, Irzyk GP, Jando SC, Alenquer MLI, Jarvie TP, Jirage KB, Kim JB, Knight JR, Lanza JR, Leamon JH, Lefkowitz SM, Lei M, Li J, Lohman KL, Lu H, Makhijani VB, McDade KE, McKenna MP, Myers EW, Nickerson E, Nobile JR, Plant R, Puc BP, Ronan MT, Roth GT, Sarkis GJ, Simons JF, Simpson JW, Srinivasan M, Tartaro KR, Tomasz A, Vogt KA, Volkmer GA, Wang SH, Wang Y, Weiner MP, Yu PG, Begley RF, Rothberg JM (2005). Genome sequencing in microfabricated high-density picolitre reactors. Nature.

[B47] Shendure J, Porreca GJ, Reppas NB, Lin XX, McCutcheon JP, Rosenbaum AM, Wang MD, Zhang K, Mitra RD, Church GM (2005). Accurate multiplex polony sequencing of an evolved bacterial genome. Science.

[B48] Hall N (2007). Advanced sequencing technologies and their wider impact in microbiology. Journal of Experimental Biology.

[B49] Scott FJ, Marchant HJ (2005). Antarctic marine protists.

[B50] Baldauf SL (2003). The deep roots of eukaryotes. Science.

[B51] Machado-Lima A, del Portillo HA, Durham AM (2008). Computational methods in noncoding RNA research. Journal of Mathematical Biology.

[B52] Telford MJ, Holland PWH (1997). Evolution of 28S ribosomal DNA in chaetognaths: Duplicate genes and molecular phylogeny. Journal of Molecular Evolution.

[B53] Dover GA (1986). Molecular drive in multigene families - How biological novelties arise, spread and are assimilated. Trends in Genetics.

[B54] Hui JHL, Kortchagina N, Arendt D, Balavoine G, Ferrier DEK (2007). Duplication of the ribosomal gene cluster in the marine polychaete *Platynereis dumerilii *correlates with ITS polymorphism. Journal of The Marine Biological Association of The United Kingdom.

